# Chromatography-Based Metabolomics as a Tool in Bioorganic Research of Honey

**DOI:** 10.3390/metabo14110606

**Published:** 2024-11-08

**Authors:** Marina Kranjac, Piotr Marek Kuś, Saša Prđun, Renata Odžak, Carlo Ignazio Giovanni Tuberoso

**Affiliations:** 1 Department of Chemistry, Faculty of Science, University of Split, Ruđera Boškovića 33, 21000 Split, Croatia; 2 Department of Pharmacognosy and Herbal Medicines, Faculty of Pharmacy, Wroclaw Medical University, ul. Borowska 211a, 50-556 Wrocław, Poland; 3 Department of Fisheries, Apiculture, Wildlife Management and Special Zoology, Faculty of Agriculture, University of Zagreb, Svetošimunska Cesta 25, 10000 Zagreb, Croatia; 4 Department of Life and Environmental Sciences, Università degli Studi di Cagliari, 09042 Monserrato, Italy

**Keywords:** bioorganic research, honey metabolites, chromatography-based metabolomics, targeted metabolomics, suspect metabolomics, untargeted metabolomics, data elaboration, origin traceability, sample preparation

## Abstract

This review presents the latest research on chromatography-based metabolomics for bioorganic research of honey, considering targeted, suspect, and untargeted metabolomics involving metabolite profiling and metabolite fingerprinting. These approaches give an insight into the metabolic diversity of different honey varieties and reveal different classes of organic compounds in the metabolic profiles, among which, key metabolites such as biomarkers and bioactive compounds can be highlighted. Chromatography-based metabolomics strategies have significantly impacted different aspects of bioorganic research, including primary areas such as botanical origins, honey origin traceability, entomological origins, and honey maturity. Through the use of different tools for complex data analysis, these strategies contribute to the detection, assessment, and/or correlation of different honey parameters and attributes. Bioorganic research is mainly focused on phytochemicals and their transformation, but the chemical changes that can occur during the different stages of honey formation remain a challenge. Furthermore, the latest user- and environmentally friendly sample preparation methods and technologies as well as future perspectives and the role of chromatography-based metabolomic strategies in honey characterization are discussed. The objective of this review is to summarize the latest metabolomics strategies contributing to bioorganic research onf honey, with emphasis on the (i) metabolite analysis by gas and liquid chromatography techniques; (ii) key metabolites in the obtained metabolic profiles; (iii) formation and accumulation of biogenic volatile and non-volatile markers; (iv) sample preparation procedures; (v) data analysis, including software and databases; and (vi) conclusions and future perspectives. For the present review, the literature search strategy was based on the PRISMA guidelines and focused on studies published between 2019 and 2024. This review outlines the importance of metabolomics strategies for potential innovations in characterizing honey and unlocking its full bioorganic potential.

## 1. Introduction

Honey is a source of numerous metabolites, some of which have been shown to have positive effects on health. In the context of bioorganic research of honey that is focused on secondary metabolites and their transformations, metabolomics is a valuable tool for exploring different metabolic profiles reflecting honey’s characteristic properties. Honey is produced by honey bees from nectar (blossom or nectar honey) or plant secretions/insect excretions (honeydew honey) [1]. Most of the secondary metabolites found in mature honey are not simply transferred from nectar to honey [2] and their formation is complex, with various factors shaping the chemical profile of honey [3]. Certain metabolites may originate from the honey bee, while some metabolites are formed or accumulated during honey ripening and are characteristic of mature honey [4].

The metabolome of honey and the differences in the metabolome between different honey types have been investigated in many recent chromatography-based metabolomics studies and are directly connected to different aspects of bioorganic research considering botanical origins (including medicinal [2,5,6], endemic [7], and traditional spice plants [8]; traditional herbal medicine [9]; etc.) honey origin traceability, entomological origins [10], and honey maturity [4], contributing to the detection, assessment, and/or correlation of various honey parameters and attributes such as geographical origin, color, quality, flavor/taste [11], adulteration/fraud [12,13], honey deterioration/spoilage [14], and bioactive potential. Flavor and aroma/odor compounds, which reflect some of the sensory properties of honey, serve as chemical markers for honey of different botanical and entomological origins [8,10], making them valuable metabolites in bioorganic research. Metabolomic analysis reveals intriguing chemical diversity among different honey types [5], while discriminatory molecules are referred to as biomarkers [15]. Some secondary metabolites such as flavonoids, phenolic acids, including cinnamic acids, and other bioactive phenolic compounds and terpenes emerged as chemical markers for botanical origin discrimination [16]. Melissopalynological analysis, which is part of the legislation for honey, is still the crucial parameter for determining its botanical origin. Combined with metabolomics, this could represent a more realistic and parallel contemporary approach [16], usually leading to higher accuracy [17]. Melissopalynological analysis is limited and challenging in some cases [18], and physicochemical indices are sometimes insufficient [12], which encourages the development of advanced analytical methods and chemometrics for authenticity assessment [19].

Unlike biogenic compounds and their natural precursors [20], some honey components (e.g., Millard reaction products [12], exogenic compounds [21]) do not reflect the native honey composition and could potentially be mislabeled as discriminatory compounds for authenticity assessment. Therefore, in metabolomic studies, large numbers of honey samples are recommended to find reliable honey biomarkers. Furthermore, finding a link between the plant, nectar, and honey allows for better confirmation of marker credibility and usefulness for traceability purposes. Nevertheless, the chemical changes that may occur during the different stages of honey formation remain a challenge. Even though there are sparse metabolomics studies that focus primarily on investigating the formation of biomarkers [2], the contribution of metabolomics to this field should not be neglected. Research into metabolite precursors, intermediates, and metabolite sources allows conclusions to be drawn about feasible biotransformation and pathways. The formation and accumulation of these metabolites play a crucial role in determining the origin and maturity of honey and correlating different honey attributes. The main issues related to honey traceability concerns are botanical, geographical, and entomological origins and maturity [4,22]. In addition, tracing the sources of compounds identified in honey can help validate biomarkers by studying their consistency in nectar/honeydew, bees, and honey, which, in turn, would contribute to the future quality control of commercial monofloral honey [9]. Research strategies include sampling during the different stages of honey formation and/or collecting and analyzing flowers, nectar, or honeydew from flowers or honey sac as well as unripe and ripe honey. This is particularly important for understanding the occurring changes and correlations between plant sources and honey as well as the metabolic connections between different metabolites and their precursors [4,23]. This approach can help researchers understand the ripening process of honey [14,24] and the molecular mechanism of its formation [4].

### Objective and Approach of This Review

Several reviews on metabolomics involving honey and other honey bee products have been published [15,17,25,26], but there is no recent detailed review explicitly focusing on honey chromatography-based metabolomics from the perspective of bioorganic research. Moreover, the chemical structures of some key metabolites are presented in this review, demonstrating the chemical diversity of the metabolites identified in the chromatographic profiles. The objective of this review is to summarize the latest metabolomics strategies in bioorganic research of honey, considering targeted, suspect, and untargeted methodological approaches, with emphasis on the (i) metabolite analysis by gas and liquid chromatography techniques; (ii) important (key) small metabolites in the obtained metabolic profiles; (iii) formation and accumulation of biogenic volatile and non-volatile markers; (iv) sample preparation procedures with an emphasis on green, non-destructive, and miniaturized methods; (v) data analysis, including software and databases; and (vi) conclusions and future perspectives of metabolomics in the bioorganic research of honey.

**Figure 1:**
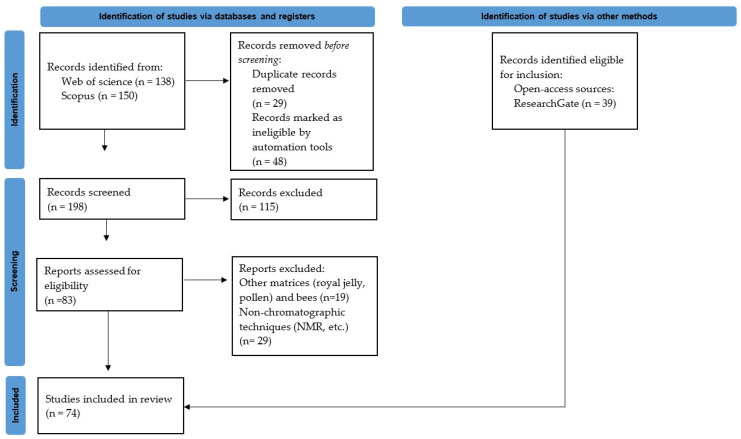
PRISMA flowchart of the included studies.

For this review, the search strategy was based on the PRISMA guidelines [27]. As the first step, online scientific databases in the field of natural sciences such as Scopus and Web of Science (WoS) were searched from 2019 to 2024 with the following search term: metabolomics honey. Some other keywords were used as part of the inclusion criteria: honey, GC-based metabolomics honey, LC-based metabolomics honey, untargeted metabolomics honey, non-targeted metabolomics honey, and targeted metabolomics honey. The inclusion criteria for the database search were only peer-reviewed and published research articles and reviews in English between 2019 and 2024 with the above term, excluding studies that did not meet these criteria. Excell was used to identify and remove duplicate publications. The methods of the selection process included screening the titles and abstracts of studies from the search results and selecting studies that met the review inclusion criteria for full-text analysis, from which data were collected/extracted. Furthermore, ReserchGate was used as an additional and valuable open-access science platform and searched according to inclusive criteria, from which relevant publications were extracted for the review. A PRISMA flowchart of the included studies is shown in Figure 1.

Bioorganic research of honey can be extended to other metabolomics applications, such as studies on the various effects of honey or honey-extracted compounds administration on metabolic pathways and metabolomic profiles in animal models, the results of which suggest the health benefits of honey. Examples of these recent studies can be found in the literature [28,29] and are briefly presented in Section 2.5.

## 2. Targeted, Suspect, and Untargeted Chromatography-Based Metabolomics

The development of an investigation by metabolomics can be driven by the typical chemical (chemical similarity), biological (functional analysis), or data-driven (statistics and machine learning) approaches. Targeted, suspect, and untargeted chromatography-based metabolomics employ several instrumental methods, including mass spectrometry (MS) [4,10,30], ion mobility spectrometry (IMS) [10,31] coupled with gas chromatography, and detectors coupled with liquid chromatography, such as high-resolution mass spectrometry (HRMS) [3], quadruple time of flight–mass spectrometry (QToF-MS) [30,32], and evaporative light-scattering detector (ELSD) [33], along with different tools for data analysis [34]. Chromatography-based metabolomics on honey use the potentialities of both gas chromatography and liquid chromatography. The integration of different chromatographic techniques ensures comprehensive information on the honey metabolome, and the combination of chromatographic and non-chromatographic instrumental techniques is also used as a more comprehensive approach [8,35]. In a study by Abd El-Wahed et al. [35], the contents of constituents such as sugars, flavonoids, and vitamins were determined by LC-MS/MS, while GC-MS was used to identify ketones and aldehydes, acids and esters, anthraquinone, phenols, hydrocarbons, and nitrogen-containing compounds [35]. The analysis of small molecules from complex biological matrices such as honey usually includes tedious sample preparation, but, recently, some new green and miniaturized technologies and methods have been proposed, allowing for comprehensive insight into the honey metabolome. Green methods have already been used in the isolation of bioactive compounds from bee products [36], but some of them are employed as a part of honey metabolomics protocols. Compared to conventional methods, green methods reduce the time required for sample preparation and solvent consumption, thereby reducing the negative impact on the environment and economy [36]. The method in metabolomic experiments should be designed in such a way that the maximum amount of information can be obtained from the available samples [37]. The choice of the green preparation method in metabolomics is therefore of crucial importance as it influences the selectivity, precision, and reproducibility of the results obtained [36].

Chromatographic methods are generally used to investigate honey traceability and authenticity [26,38]. Chromatographic analysis of honey headspace and extracts or native honey samples provides metabolite profiles with the possibility of revealing new or previously known key metabolites, such as potential biomarkers (often major compounds), biologically active compounds (BACs) (e.g., health-promoting or even toxic compounds), bee- and plant-derived compounds (phytochemicals/phytogenic components), and flavor and aromatic compounds. In contrast to targeted metabolomics, which aims to determine already known metabolites of interest, untargeted metabolomics enables the detection and/or identification of novel compounds and reveals specific metabolite patterns of different honey types (metabolite fingerprinting). Both an untargeted approach, which focused on clustering and the identification of markers, and a targeted analysis, which allowed for the quantification of markers, were used to distinguish honey from *Apis cerana* and *Apis mellifera* bee species, i.e., to determine its entomological origin [10]. The targeted identification and quantification of honey constituents is particularly valuable in the case of metabolites that are characteristic of many honey varieties but that are accumulated and increased differently in some honey types. Karabagias et al. [39] applied machine learning algorithms to semiquantitative honey volatilome data and introduced a Karabagias–Nayik index (R_ch_) to control citrus honey authentication. The complementarity of targeted, suspect, and non-targeted metabolomic approaches was highlighted in the work of Koulis et al. [40], in which twelve additional bioactive compounds in Greek honey were identified and semi-quantified by suspect screening. Dallagnol et al. [41] characterized honey from the honey bee *Tetragonisca fiebrigi* using metabolomics in combination with microbiological and physicochemical analyses and found that flavonoids and phenylethylamides are crucial factors for its antimicrobial properties. For the first time, a metabolomic profiling study was conducted to differentiate the anticancer potential of honey from Malaysia and New Zealand [42]. Shamsudin et al. [30] used an untargeted metabolomic approach for the first time to determine potential metabolites associated with antioxidant activity in stingless bee honey. Furthermore, an untargeted metabolomic methodology was used to identify pharmaceutical honey volatile metabolites (PHVMs) [43]. Of course, the differences in the chemical compositions across different honey matrices such as blossom honey, honeydew, and processed/heated honey can be a valuable tool for honey differentiation and the detection of specific key metabolites, especially considering the disadvantages of melissopalynological analysis (e.g., honey filtration). Nevertheless, the differentiation of some honey types based on an untargeted metabolomic approach can be limiting, as similar chemical profiles with non-specific chemical markers may occur. Therefore, quantification of the targeted metabolites is an additional step to overcome this limitation and draw conclusions. Kynurenic acid, for example, is a metabolite that has been identified in metabolic profiles of different honey types but is known as a chemical marker for *Castanea* spp. honey due to its high content in this honey. In addition, the difference in kynurenic acid content served to differentiate Shennongjia and Yunnan honey, with honey from the Shennongjia region being significantly enriched [44]. Kynurenic acid has also been identified as a marker for *Apis meliffera* honey [45], but further metabolomics study has shown that high kynurenic acid content is not species-specific [44]. Therefore, if conclusions are to be drawn from the chemical analysis of honey metabolites and the identification of specific metabolites, the numerous factors such as climate and region (e.g., high-altitude adaptation [44], storage conditions [46]) that influence the chemical composition of honey should be taken into account. The chromatography-based metabolomic approaches mentioned above are valuable for discovering the diversity of honey metabolites and their associated attributes. Further case studies illustrating the practical applications of these chromatography-based metabolomics approaches are discussed in the following sections.

### 2.1. Gas Chromatography-Based Metabolomics

#### 2.1.1. Exploring Metabolic Profile Diversity: Identification of Key Metabolites

Metabolomic strategies based on the analysis of volatile organic compounds (VOCs) or volatile metabolites (VMs) of honey have been efficiently used to discriminate honey of different floral, geographical, and entomological origin (e.g., honey of *A*. *cerana* and *A. mellifera*), identify reliable markers [7,13], and link the honey type and its composition with its characteristic properties.

Wang et al. [47] used untargeted imaging of volatile compounds obtained from headspace gas chromatography–ion mobility (HS-GC-IMS) in combination with chemometrics and markers response to discriminate winter honey (honey collected during winter from *Schefflera actinophylla* (Endl.) Harms and wild *Eurya* spp.) from *sapium* honey (honey collected in summer from *Sapium sebiferum* (L.) Roxb) and found benzaldehyde dimer and phenylacetaldehyde dimer to be markers for winter honey and phenylethyl acetate dimer to be a marker for *sapium* honey. In another study, non-targeted HS-GC-IMS-based metabolomics including orthogonal partial least squares-discriminant analysis (OPLS-DA) was proposed for the characterization of honey botanical origin [31], while quantitative analysis additionally revealed higher concentrations of valeraldehyde and hexanal in albaida honey and a higher content of 6-methyl-5-hepten-2-one in orange blossom honey. Furthermore, different varieties of honey botanical sources within a genus influence both the VOC concentrations and sensory profiles [48].

Sichilongo et al. [7] reported that some of the VMs identified in commercial polyfloral honeys (cyclopentadecanone, α-methyl-α-[4-methyl-3-pentenyl]oxiranemethanol, eicosane, tricosane) and unprocessed polyfloral honeys (e.g., *trans*-linalool oxide) are major components of conifers, flowering plants, and other common species of the respective geographical area from which the honey originates. In this comprehensive metabolomic study, the authors combined the Automated Mass spectral Deconvolution and Identification System (AMIDIS), *Metab* R software package (R Development Core Team, 2018; https://www.R-project.org/, accessed on 27 September 2024), and statistical analysis software MINITAB version 14 to classify the botanical and geographical origins of selected commercial and unprocessed honeys using GC–MS untargeted metabolomics of volatile components [7]. The characteristic classes of organic compounds and the number of metabolites were reported according to the type and origin of the honey, with a lower number of metabolites observed in commercial honey compared to raw honey, which was attributed to the loss of some VOCs during honey processing [7]. In a study by Karabagias et al. [39], machine learning algorithms were applied to semiquantitative data of citrus and other types of honey in which different amounts of specific volatile compounds, including lilac aldehyde D, dill ether, 2-methylbutanal, heptane, benzaldehyde, α,4-dimethyl-3-cyclohexene-1-acetaldehyde, and herboxide (isomer II), contributed to discriminate citrus honey according to geographical origin. In another study, both clustering and discriminant analyses were applied in combination with a volcano plot to classify honey from different regions, and exclusive potential volatile markers were identified, i.e., hexadecane, cyclodecane, octadecane, (1-butylheptyl)-benzene, and (1-propyloctyl)-benzene [49]. Some chemical classes and organic compounds identified in metabolic profiles are known for their biological/pharmacological activities, presenting desirable components of honey that usually give it additional value and specific properties useful for human health. For instance, the organosulfur compound sulfonylbis-metahane, identified in polyfloral commercial honey from Botswana, is reported to have pharmacological activities in humans [7]. Untargeted metabolomics reveals potential metabolites related to antioxidant activity besides the well-studied polyphenolic antioxidants, with pinitol, mannitol, gluconic acid, and myo-inositol identified using GC-MS for stingless bee honey as having a greater impact on antioxidant activity [30]. Zhang et al. [24] successfully applied principal component analysis (PCA) based on antioxidant parameters and volatile components to determine honey maturity. It is interesting to point out that the antioxidant capacity of honey increases as it matures [24]. Karabagias et al. [43] applied a non-targeted metabolomic methodology using headspace solid-phase microextraction and gas chromatography–mass spectrometry (HS-SPME/GC-MS) to identify pharmaceutical honey volatile classes/metabolites (PHVMS) in different types of Greek honey. Among the PHVMs, which included terpenes, norisoprenoids, benzene derivatives/phenolic volatiles, and other compounds, the newly identified norisoprenoid 3,4,6,6-tetramethylbicyclo[3.2.1] oct-3-ene-2,8-dione contributed to the aroma of Greek *Arbutus unedo* L. honey. It has already been pointed out that VOCs play a significant role in honey aroma [50] and are closely related to honey’s botanical origin [43], forming the basis for honey differentiations in metabolomic studies [7]. In a study by Kang et al. [51], metabolomics was combined with sensory analysis to determine correlations between the chemical profile and sensory quality of honey. Volatiles with floral notes (e.g., decyl formate) were preferred by consumers, while others with off-flavors (e.g., 2-methylbenzofuran) were not preferred by them. Key flavor compounds can be found among volatile and non-volatile honey constituents [51], which is why the combination of GC and LC chromatography is employed in this kind of studies. Some authors combined GC- and LC-based metabolomics approaches to further accurately quantify and/or verify the identified key compounds in other honey types. Examples of these studies [4,18,33,35] are also discussed in this review, and LC analyses are presented separately in Section 2.2. Some other metabolomics studies on honey volatiles have also combined untargeted metabolomics with other approaches, e.g., targeted metabolomics [10]. Wang et al. [10] successfully combined untargeted and targeted metabolomics analyses in combination with PCA, OPLS-DA, and VIP analysis based on volatile compounds to distinguish honey from *A. cerana* and *A. mellifera* for the first time. Targeted analysis confirmed 1-nonanol, 1-heptanol, and phenethyl acetate as *A. cerana* honey markers, while benzaldehyde, heptanal, and phenylacetaldehyde were determined to be markers for *A. mellifera* honey. These species markers with specific odor characteristics were not influenced by floral or geographical origin [10], and, indeed, phenylacetaldehyde and acetaldehyde are already known to be common aromatic compounds present in different honey types. Later, Xiaotong Liu et al. [52] reported hydroxy fatty acids (bee-derived components) as novel markers for the identification of *A. cerana* honey and *A. mellifera* honey, where 8-hydroxyoctanoic acid and 3,10-dihydroxydecanoic acid could be used as markers for the accurate identification of the entomological origin of honey [52]. Sharin et al. [53] applied PCA, PLS-DA, and machine learning (support vector machine) to a combination of dataset consisting of physicochemical properties and GC-MS volatile profiles to differentiate Malaysian stingless bee honey from different entomological origins (*Heterotrigona bakeri, Geniotrigona thoracica*, and *Tetrigona binghami*). The authors found that profiles of *H. bakeri* and *G. thoracica* honey were close to each other but clearly separated from *T. binghami* honey, which was characterized by a high abundance of 2,6,6-trimethyl-1-cyclohexene-1-carboxaldehyde, 2,6,6-trimethyl-1-cyclohexene-1-acetaldehyde, and ethyl 2-(5-methyl-5-vinyltetrahydrofuran-2-yl)propan-2-yl carbonate. Copaene was proposed as a chemical marker for *G. thoracica* honey [53].

Table 1 lists examples of key metabolites identified by various metabolomics strategies in different honey types.

#### 2.1.2. Exploring Origin, Formation, and Accumulation of Biogenic Volatile Key Metabolites

Montaser et al. [5] performed a detailed metabolomics analysis of the honey bee and its products (honey, royal jelly, and bee bread) of three medicinal plants (marjoram, trifolium, and citrus) using GC-MS with headspace analysis followed by multivariate analysis. Some volatile compounds appeared in the bee and its product, e.g., *trans*-beta-ionone-5,6-epoxide was identified in the citrus bee and its honey. In a study by Leoni et al. [18], the correlation between raspberry pollen and secondary volatile and non-volatile metabolites was investigated by melissopalynological analyses and untargeted metabolomics. Among the volatile organic compounds, nicotinaldehyde was present in all honey samples and showed a significant correlation with the pollen count of *Rubus idaeus* L. [18].

Significant differences were found in the HS-SPME-GC-MS generated volatile components of buckwheat honey at different levels of maturity, with esters and alcohols dominating at lower maturity levels and aldehydes and acids at higher maturity levels [54]. Sha Yan et al. [33] profiled trace oligosaccharides at different ripening stages and, using a GC-MS-based metabolomics strategy, found that turanose content is elevated in mature acacia honey samples, making it a potential marker for acacia honey maturity. Zhang et al. [24] identified headspace VOCs nonanal, benzaldehyde monomer, and benzaldehyde dimer as potential maturity indicators for the identification of mature rape honey. A similar approach may be useful to detect the contamination of honey with osmotolerant yeast, naturally present in honey. HS-SPME-GC-MS combined with PCA and OPLS-DA allowed for the differentiation of *Zygosaccharomyces rouxii*-contaminated jujube honey from uncontaminated honey based on changes in volatile profiles in mature and immature honey. Undecanal, methyl butyrate, methyl 2-nonenoate, methyl hexanoate, and 2-methyl-3-pentanone were identified as markers of jujube honey contaminated with *Z. rouxii*, while methyl heptanoate, 2,6,10-trimethyltetradecane, and heptanal were identified as potential markers for immature jujube honey contaminated with *Z. rouxii* [14]. Another study based on an analysis of volatile metabolites based on GC-FID/MS analyses of HS-SPME and dehydration homogeneous liquid–liquid extraction (DHLLE) in combination with PCA allowed researchers to find compounds related to honey fermentation by *Saccharomyces cerevisiae* as well as thermal treatment of the honey before fermentation. The obtained meads, regardless of botanical origin, contained trytophol related to yeast metabolism and higher levels of aliphatic acids and esters but fewer aliphatic hydrocarbons than honey. Boiled meads contained more aliphatic alcohols and acids and unboiled meads contained more aliphatic hydrocarbons and esters. This research identified known chemical markers of botanical origin that remained unchanged in the meads [55]. There are examples of compounds found in honey that also originate from other sources, such as the fluorinated natural product found in unprocessed honey, 1-fluoro-4-methylbenzene, which is thought to be a product of fungal co-metabolism of toluene by *Cunninghamella echinulata* and *Aspergillus niger* and other forms of bacteria [7].

### 2.2. Liquid Chromatography-Based Metabolomics

#### 2.2.1. Exploring Metabolic Profile Diversity: Identification of Key Metabolites

Díaz-Galiano et al. [56] used a UHPLC-QToF-HRMS-based metabolomics approach to clearly distinguish manuka (*Leptospermum scoparium*) honey from other types of monofloral honey and from adulterated manuka honey samples. In their work, previously reported manuka honey compounds (leptosperin, lepteridine, 3,4,5-trimethoxybenzoic acid, 3-hydroxy-1-(2-methoxyphenyl)penta-1,4-dione, 2′-hydroxyacetophenone) along with newly elucidated structures found for the first time in manuka honey, in particular, leptosperinic acid, leptosperin triglycoside, methyl syringate dimer, methyl syringate trimer, acetosyringone, α-hydroxy-2-methoxy-γ-oxobenzene butanoic acid, and paeonol, were identified as exclusive manuka markers. In a study by Guo [57], manuka honey was differentiated from other types of honey for the high contents of 4-methoxyphenyllactic acid and *p*-hydroxyhydrocinnamic acid, indicating the effects of botanical and geographical origins on phenolic profiles. Phenolic compounds, along with triterpenes, were identified as potential chemical markers of heather honey using the UHPLC-HRMS untargeted metabolomics workflow including statistical analysis [16]. Most of the potential markers from the above chemical classes have not yet been reported in this Greek honey, including some key bioactive metabolites such as the iridoid glycosides catalpol and aucubin and ganolucidic acid B, which also belongs to the terpenoid family [16]. The same author conducted a UHPLC-HRMS metabolomics study integrating GC-MS and HPLC-PDA-ESI/MS with melissopalynological analysis [3]. The study focused on the differentiation of the three types of orange blossom honey (from Italy, Greece, and Egypt) according to geographical origin. For statistical analyses, UHPLC-HRMS data were analyzed applying PCA and OPLS-DA and evaluated based on VIP scoring. Italian honey showed a higher level of flavonoids, while, in honey from Greece, terpenoids and iridoids were more abundant than flavonoids (except hesperidin). On the other hand, Egyptian honey was characterized by suberic acid and fatty acid ester derivatives. These compounds may be potentially associated with citrus varieties and the local indigenous flora [3]. The compounds structurally related to volatile nicotinaldehyde identified in the chemical profile of red raspberry (*Rubus idaeus* L.) honey were nicotinamide, nicotinic acid, and nicotinyl alcohol, which were present in all samples and correlated with the pollen count of *R. idaeus* [18]. Using targeted and untargeted metabolomics analyses, N_1_, N_5_, N_10_-(*E*)-tricoumaryl spermidine was identified as the plant-derived characteristic compound in *Triadica cochinchinensis* honey (TCH) [58]. Targeted and non-targeted metabolomic workflows were also developed and applied by Koulis et al. [32] using a UPLC–QToF-MS method, screening phenolic compounds in different types of honey originating from Greece and Poland. Later, the same authors [40] investigated phenolic compound profiles using complementary metabolomic workflows focusing on reputable Greek honey varieties from five different botanical sources. In addition, Zhao et al. [59] applied a combined untargeted and targeted UPLC-Q-TOF-MS/MS-based approach to identify and quantify markers of monofloral honey from *Astragalus membranaceus* var. *mongholicus* Hsiao. In the study, calycosin and formononetin were identified as reliable chemical markers for this honey, which were also identified in the *Astragalus membranaceus* var. *mongholicus* Hsiao plant [9]. To characterize monofloral honey (MSH), Zhao et al. [59] applied non-targeted UHPLC/Q-TOF-MS-based metabolomics to screen and compare the components of safflower honey and flowers and found safflomin A to be a novel reliable marker. Montoro et al. [11] applied a chemometric model to data obtained by liquid chromatography coupled with high-resolution tandem mass spectrometry in negative ion mode using a mass spectrometer with an electrospray source coupled to a hybrid high-resolution mass analyzer (LC-ESI/LTQ-Orbitrap-MS data) to detect the bitter-tasting compounds in strawberry tree honey (*Arbutus unedo*). The phenols sakuranin and kurarinone, suspected to be potential sensory biomarkers, were found in the more bitter honey fraction, with unedone being the most abundant, a metabolite particularly responsible for the bitter taste [11].

Metabolomics tools, including multivariate analysis, were applied to concatenated LC-HRMS and NMR datasets to provide an intensive metabolite profile of Malaysian honey samples (higher sugar and polyphenol content) and New Zealand honey samples (higher concentration of low-molecular-weight lipids). Putative mild antioncogenic compounds against the breast cancer cell line ZR75 were identified in Malaysian honey, such as gingerdiol, 2-hexylphenol-*O*-β- d -xylopyranoside, plastoquinone, tropine isovalerate, plumerinine, and 3,5-(12-phenyl-8-dodecenyl)resorcinol, together with several phenolic esters and lignans [42]. An untargeted liquid chromatography–mass spectrometry (LC-MS) metabolomics approach was used to identify antioxidant compounds in unifloral stingless bee honey, which mainly included alkaloids and flavonoids [60]. In a study by Dallagnol et al. [41], six new flavonoids from stingless bee honey, namely, quercetin 3,4′-dimethyl ether, pachypodol, jaceoside, irigenin trimethyl ether, corymboside, and chrysoeriol 7-neohesperidoside, were annotated by liquid chromatography with tandem mass spectrometry (LC-MS/MS) analysis and supported by metabolomic tools. Another compound from the flavonoid class, isorhamnetin 3-*O*-neohesperidoside, was found to be the characteristic substance that distinguishes the rare *Amomum tsao-ko* Crevost et Lemari’e honey from other types of honey [8].

Wang et al. [45] proposed an untargeted strategy based on UPLC/ESI Q-Orbitrap MS followed by targeted metabolomics based on ultrahigh-performance liquid chromatography coupled with triple quadrupole tandem mass spectrometry (UPLC-MS/MS) to demonstrate 3-amino-2-naphthoic acid and methyl indole-3-acetate as markers of *A. cerana* honey (present in higher amounts in *A. cerana* honey) and kynurenic acid as a marker of *A. mellifera* honey. In a study by Guo et al. [57], *A. cerana* honey and *A. mellifera* honey were differentiated on the basis of the phenolic profiles, with caffeic acid and pinobanksin ester derivatives being rarely present and at lower levels in *A. cerana* honey than in *A. melliffera* honey. In a study by Rivera-Perez et al. [46], UHPLC-Q-Orbitrap-HRMS-based metabolomics was used to discriminate among commercially available monofloral (eucalyptus, orange blossom, and rosemary honey) and multifloral-labeled honey from the Spanish market. Up-accumulated key metabolites from diverse classes of organic compounds were found in the honey samples, including amino acid L-phenylalanine and trisaccharide raffinose for rosemary honey and alkaloid trigonelline for multifloral honey.

Table 2 lists examples of key metabolites identified by various metabolomics strategies in different honey types.

#### 2.2.2. Exploring Origin, Formation, and Accumulation of Biogenic Non-Volatile Key Metabolites

**Figure 2:**
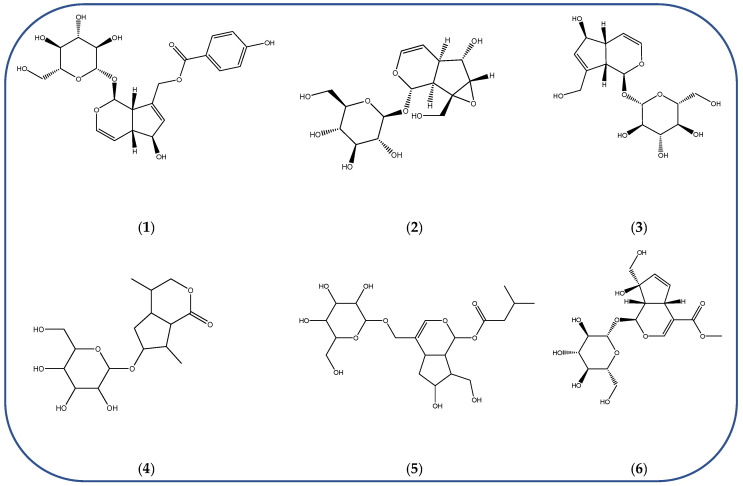
Chemical diversity of iridoids/iridoid glycosides accumulated in chaste honey, agnuside (**1**); “Anama” honey, catalpol (**2**) and aucubin (**3**); Greek citrus honey, nepetaside (**4**) and patrinoside (**5**); and thyme honey, gardenoside (**6**).

Understanding the formation of the biomarkers responsible for the chemical diversity and unique properties of honey can help to control and obtain high-quality products rich in natural bioactive compounds [2]. Sha Yan et al. [2] investigated the formation mechanism and accumulation of markers from nectar to mature honey using a comparative metabolomics approach [4] and pointed to the pivotal role of honey bees in this process. Organic acids and phenolic compounds were the main small molecules identified in chaste honey extracts with agnuside (Figure 2 (**1**)) and were assigned as reliable chemical marker of chaste honey and type of iridoid glycoside with anti-inflammatory properties. Agnuside was employed in the quality control of the *V. negundo* Linna. Var. *heterophylla* (Franch.) Rehd. medicinal plant. The nectar contained low agnuside levels, and dehydration and ripening substantially increased the agnuside levels in chaste honey. Besides agnuside, they explored the distributions of salicylic acid and *p*-hydroxybenzoic acid and their predominant derivatives over diverse segments of the chaste plant (stem, leaf, flower, nectar, honey) and displayed the chemical correlations and formations of the identified glucosylated phenolic biomarkers (4-(*β*-D-glucosyloxy) benzoic acid, 1-(4-hydroxybenzoyl) glucose, and 6-*O*-(*p*-hydroxybenzoyl) glucose. Free *p*-hydroxybenzoic acid was detected in chaste plant and could be converted into glucosylated phenolic biomarkers. *p*-Hydroxybenzoic acid was one of antibacterial metabolites of *Castanopsis* honey [62] and, through targeted analysis, was found in high concentrations in buckwheat honey [32]. All the abovementioned chemical biotransformation included glycosyilation, isomerosation, and methylation reactions. In addition, these authors [2] found that 4-(*β*-D-glucosyloxy)-benzoic acid is not present in chaste nectar and that the conversion and accumulation of this chemical marker is affected by the activity of honey bees during the collection and processing of nectar into mature honey. The changes occurring in honey may be useful to evaluate honey quality in terms of its maturity. Sun et al. [4] studied the molecular mechanisms of mature honey formation, applying a UPLC-QTOF-MS-based metabolomics approach (combined with PCA and OPLS-DA) to study metabolites of stomach honey, immature honey, and mature rapeseed honey samples. Mature honey was found to represent a metabolic profile distinct from that of immature honey [4,63], characterized by higher levels of decenedioic, myristic, myristoleic, and behenic acids, and decenedioic acid as a bee-originated fatty acid was also verified in other honey types [4]. Guo et al. [49] showed a higher accumulation of the major polyphenolic components in mature rapeseed honey using targeted metabolomic analyses, where some such as kaempferol, apigenin, pinocembrin, and 3-(3,4-dimethoxyphenyl)-2-propenoic acid were only detected in mature honey. In a study by Liu et al. [58], two patterns of the honey maturation process were identified based on 723 metabolite signature transformations. The first pattern was that the content of plant-derived compounds with a strong reducing effect, such as spermidine, flavonoids, and their derivatives, was reduced. The second pattern was that the maturation process of honey was accompanied by the formation of lactone glycoside analogs and organic acids, which was probably facilitated by the enzymatic transformation of enzymes secreted by bees [58].

Gao et al. [64] revealed significant chemical variation between nectars and their corresponding honey, detecting hesperidin in all nectars and hesperetin in all honey samples, suggesting the latter as a suitable marker for the floral origin of *Citrus* honey [64]. Knowledge of the formation of biomarkers can be useful to explain their levels in honey [3] and consider the factors that influence their accumulation, e.g., the effects of hydrolytic enzymes (glucosidases) and oxidative enzymes (glucose oxidase) [46]. Gao et al. [64] concluded that the amount of hesperidin in honey decreased because of the hydrolyzation of hesperidin to hesperetin by the action of enzymes in bee saliva, while, in a study by Kasiotis et al. [3], the accumulation of hesperidin was observed to be characteristic of Greek citrus honey samples. Compared to monofloral honey, including orange honey, the Amadori compounds *N*-(1-deoxy-1-fructosyl)isoleucine and *N*-(1-deoxy-1-fructosyl)phenylalanine were identified as highly accumulated metabolites in multifloral commercial honey from Spain. It is known that these types of compounds are formed in honey during processing or prolonged storage. Nevertheless, these results indicate a potentially greater availability of Maillard reactants (reducing sugars and amino acids) in multifloral honey [46].

The integration of melissopalynological analysis in metabolomics studies, which provides detailed information on the local indigenous flora surrounding hives, contributes significantly to tracing the origins of the metabolites identified in the respective honey metabolome. In addition, the influence of indigenous flora is important when studying the effects of different geographical origins on the metabolome of the same honey types, [3] while annotations of key metabolites in honey can be additionally substantiated by the occurrence of the corresponding plant source [3]. In studies by Kasiotis et al. [3,16], along with markers of botanical origin, key metabolites found in metabolomic profiles of orange blossom, heather, and thyme honey were connected with other Mediterranean plants identified via melissopalynological analysis and previously reported for Mediterranean plant sources, e.g., bioactive flavonoid dihydrokaempferol (DHK) was connected to the Cactaceae family [3], the rare terpenic molecule secologanate was connected to *Dendrobium* species [3,16], and the bioactive iridoid gardenoside originated from Gardenia fruits [16]. The iridoid glycosides were previously identified in Greek honey [16], while, in a study by Kasiotis et al. [3], they were detected for the first time in Greek citrus honey and attributed to plants from the Boraginaceae family (rare iridoid nepataside) and *Valeriana* species (iridoid glycoside patrinoside) from the Mediterranean region. The maturation process of honey [58] was accompanied by the formation of lactone glycoside components. Iridoid glycosides represent an under-researched chemical family that represents an interesting source of potentially bioactive compounds. Examples of iridoids/iridoid glycosides found in different types of honey are shown in Figure 2.

**Figure 3:**
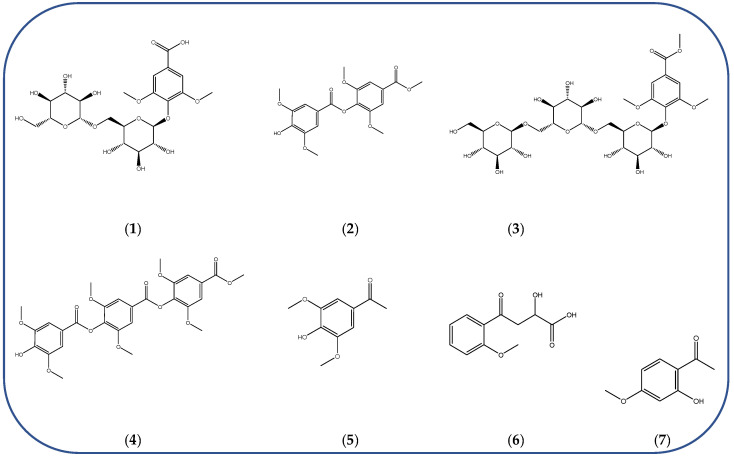
Newly elucidated structures of the markers exclusive to manuka honey: leptosperinic acid (**1**), methyl syringate dimer (**2**), leptosperin triglycoside (**3**), methyl syringate trimer (**4**), acetosyringone (**5**), α-hydroxy-2-methoxy-γ-oxobenzene butanoic acid (**6**), and paeonol (**7**).

Some key constituents were identified for the first time in some types of honey and in sources different than plants and beekeeping matrices, e.g., ganolucidic acid B was identified in Greek heather honey (“Anama” honey) and isolated from the fungus *Ganoderma lucidum* [14]. Key metabolites already identified and the knowledge of their structural features may contribute to the elucidation of new chemical markers, just as further research on possible combinations of identified secondary metabolites may contribute to the elucidation of additional markers [56]. Most of the identified exclusive manuka markers (summarized in Figure 3) are derivatives or analogs of the previously identified manuka markers methyl-syringate and leptosperin [56], for which several glycosylated and demethylated analogs of leptosperin were elucidated (Figure 3, compounds (**1**) and (**2**)), methyl-syringate condensation products (Figure 3, compounds (**3**) and (**4**)), and other analogs such as acetosyringone (Figure 3, compound (**5**)). The remaining structures displayed in Figure 3 can also be viewed as reaction product (compound (**6**)) or analogue (compound (**7**)) of other well-known compounds occurring in manuka honey [56]. After manuka honey, Sidr honey is reported to be the most popular honey [35]. In a comprehensive metabolomics approach by Abd El-Wahed et al. [35], a valuable global natural products social molecular networking (GNPS) workflow was applied as part of an investigation of Sidr honey. Molecular networking was created for honey samples, where the parent ions identified in the GNPS molecular network were represented by the triangle nodes [35]. Furthermore, GNPS workflow was employed to explore the chemistry of stingless bee-plant symbiosis and the antimicrobial properties of stingless bee honey. Both studies emphasized the importance of flavonoids, and Dallagnol et al. [41] also reported that honey samples missing antimicrobial activity also lacked flavonoids. The application of this metabolomic tool can help in metabolite discovery and can be a valuable and interesting tool in bioorganic research, as the method takes into account potential functional relationships representing the most reliable metabolite–metabolite interactions within the network [65].

### 2.3. Pre-Analytical Sampling Design and Sample Manipulation

Pre-analytical factors can strongly influence the subsequent chromatographic analysis and results. For this reason, each project requires a specific setup and should be carefully planned. First of all, regarding the honey samples, different types of sampling designs can be used in this context. The number of authentic honey reference samples selected with regards to the characteristic attributes to be investigated (e.g., honey from each flora type [60]) should be sufficiently large (e.g., a total of 89 honey samples) [31] and obtained from trustworthy sources such as local apiaries [2,31] and professional beekeepers [43] or collected from large orchards [10]. This step is fundamental because the collected samples are a subset of a larger group of samples (population) from which conclusions have to be drawn. In this way, authentic sets of honey samples could be established for analysis as training set and external test set samples [66]. In work by Wang et al. [45], additional authentic samples were set to test the accuracy and content of the selected authentication markers in honey with different entomological origins [45]; while the applicability of the method can be demonstrated by analyzing samples of unknown botanical origin [31]. When researching rare types of honey, it is difficult to obtain a wide number of authentic honey samples [18] and find chemical markers for its authentication [8], so collaboration in characterizing the available samples from different researchers is advisable [18]. Usually, sample authenticity and quality are ascertained by melissopalynological analysis, sensory analysis, and determining physicochemical parameters.

Prior to sample preparation and analysis, samples are usually refrigerated in screw-top jars or airtight jars at 4 °C [10,43] or sub-zero temperatures [14,16,24] to avoid the volatilization and decomposition of volatile compounds [10] and later tempered at room temperature [31]. In addition, crystallized honey samples are heated to 40 °C in a water bath to homogenize them or reduce the moisture content, i.e., dehumidify them [10,30,40].

Usually, the purification/clean-up of samples and extraction need to be done prior to LC or GC separation in order to concentrate the organic metabolites of interest or reduce interfering compounds (e.g., sugars and proteins). Due to the high viscosity of honey, the first step of the sample preparation protocol for LC-based metabolomics analysis usually consists of the dissolution/dilution of honey samples in ultrapure or acidified water [3,16,66]. Thereafter, optimized conventional liquid–liquid extraction (LLE) [32] or an alternative and novel method such as the simple QuEChERS (Quick, Easy, Cheap, Effective, Rugged and Safe) method can be applied, as well as sugaring-out assisted liquid–liquid extraction (SULLE) [58,67]. SULLE offers a higher extraction efficiency compared to conventional LLE [67] and represents a simple and fast extraction process. Similar to QuEChERS, only small volumes of organic solvents are used compared to conventional extraction methods [36,67]. An ultrasound-assisted and miniaturized extraction process can also be applied [4] as well as other modified and optimized LLE involving steps such as stirring/vortexing/shaking and centrifugation [45,65,68]. Furthermore, in order to concentrate and extract compounds of interest (e.g., active substances [63]), in some cases, compounds are eluted through solid-phase extraction (SPE) cartridges [9,16,41] or polyaromatic adsorbent resin (Amberlite XAD-2) [60]. In general and simplified terms, the resulting eluates/extracts are filtered [41] and/or dried/concentrated using a nitrogen stream [32,63], in vacuo on a rotary evaporator [42], or in a concentration vacuum centrifuge [59] and redissolved/reconstituted in an appropriate solvent and filtered [9] before injection into the chromatography system. Concentrated [60] or water-soluble extracts can be freeze-dried [42]. Another practice in sample preparation/manipulation is the preparation of quality control (QC) samples by combining equal amounts of each sample or extract obtained [13,32,40]. QC samples are important for monitoring instrument sensitivity and stability as well as for correcting metabolomic data [44]. In addition, a blank sample obtained by applying the preparation procedure without adding a honey sample can be used [58] or, simply, ultrapure water can be subjected through procedural steps [32,40].

**Figure 4:**
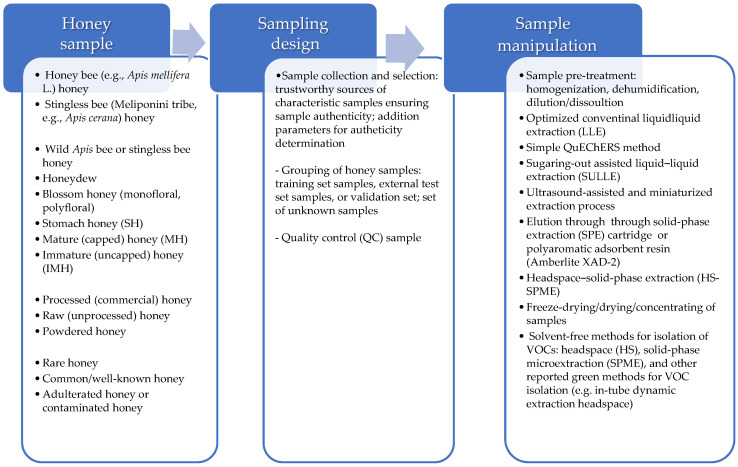
Schematic representation of sample types, sampling design, and sample manipulation prior to analysis by GC and LC chromatographic methods in bioorganic research.

Metabolomics helps in reducing clean-up steps as efforts are directed towards finding suitable methods with minimal sample manipulation to obtain a comprehensive chemical profile of the sample and avoid matrix alterations/artifacts or contaminants. Environmentally friendly analytical methods involving less sample manipulation are reported, using different sample introduction systems such as headspace (HS) [24,31], enabling the direct volatilization of scent compounds [5,47], or solid-phase microextraction (SPME) as another headspace sampling method [28,29]. Different fiber coatings are available for VOC extraction in the SPME method, with the highest extraction efficiency achieved with mixed-phase DVB/CAR/PDMS coating for cocoa honey samples [48]. Besides this direct, solvent-free isolation of honey volatile metabolites from honey native samples, other methods for VM isolation are reported. In some studies, alcoholic honey solutions were prepared and subjected to vacuum drying [33] or the honey samples were subjected to freeze-drying [30], and, in both cases, derivatization was performed prior to GC–MS analysis [30,33]. Liquid–liquid extraction (LLE), usually assisted by vortex and/or ultrasound, with a reduced amount of an organic solvent such as n-hexane and shortened extraction time, is also used to isolate volatile components [49]. Apart from the abovementioned methods, there are other recently reported green methods of sample preparation that have been successfully applied to honey VOC isolation and may be suitable for metabolomics research such as in-tube dynamic extraction headspace (ITEX-DHS) [69]. Figure 4 shows a representation of sample types, sampling design and the sample manipulation prior to chromatographic GC and LC analysis.

### 2.4. Data Analysis Workflow: Software and Data Elaboration

As previously discussed, for the development of metabolomics, besides the analytical investigation, it is necessary to manage different chemometrics (multivariate statistical analysis) approaches. Several crucial aspects should be evaluated: the ability to process the raw spectral data, the use of statistical analysis to find significantly expressed metabolites, the ability to connect to metabolite databases to identify metabolites, the bioinformatics analysis and visualization of molecular interaction networks, and the ability to integrate and analyze multi-omics data.

Chemometrics is indeed an extremely important tool that enables the processing of large amounts of data through multivariate analysis and machine learning techniques, an essential step for the analysis and extraction of relevant information from extremely complex metabolic systems, typical of “omics” studies. This process of sifting through massive amounts of data to identify hidden trends or patterns is often called “data mining”. The combined metabolomics and bioinformatics tools have contributed to the discovery of numerous bioactive compounds from honey and other beehive products. Recently, researchers have been looking with interest to more sophisticated approaches that involve the application of artificial intelligence (AI) tools [70]. AI techniques, particularly machine learning and data analysis algorithms, are being integrated with mass spectrometry to enhance the interpretation and analysis of the complex data generated by these instruments. They can also be used for the optimization of instrument parameters. The constant implementation of those models draws inspiration from every research field. For instance, the Gestalt principles are commonly applied in art and design, but lately represent a further set of rules describing how humans perceive and interpret visual information [71,72]. AI-based solutions for chromatographic data processing for honey are not being applied yet, but they look promising as tools, especially for 2-D applications (GCxGC and LCxLC).

**Figure 5:**
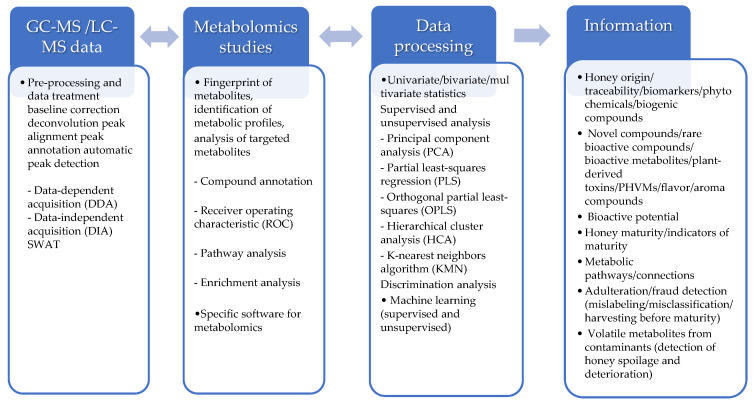
Schematic representation of the analysis of metabolomics data generated with GC and LC chromatographic methods in bioorganic research with the information obtained.

Figure 5 reports the basic steps in the data analysis workflow, from data treatment to obtaining the final information. First of all, the two most popular acquisition techniques employed in mass spectrometry are data-independent acquisition (DIA) and data-dependent acquisition (DDA). DIA captures all of the fragment ions within a predetermined *m/z* range, with the detection and quantification of all analytes in a sample. DDA selects specific ions to fragment based on their level of abundance or *m*/*z* value. SWATH-MS is a specific variant of the DIA method and is a valuable tool for large-scale proteomics. Usually, after data acquisition by LC-MS and GC-MS, data are processed (pre-processing and data treatment, baseline correction, deconvolution, and peak alignment; data-dependent and data-independent acquisition methods) and elaborated by chemometric tools with appropriate software (classification, statistical validation, identification of metabolites) and metabolic pathway analysis by databases. The selection of the proper tool depends on the metabolomics approaches used to elucidate complex matrices of natural products (fingerprinting of metabolites, identification of metabolic profiles, or analysis of targeted metabolites).

Typical MS chromatographic dataset consist of time, *m/z*, and intensity parameters (features), and, for reliable detection and quantification, data (pre)processing is a fundamental step that eliminates or reduces variations from data acquisition to statistical analysis. Background correction and peak detection benefit from the use of specific algorithm and Autoencoder is a robust tool [73]. Generally, monitoring and controlling data quality are crucial points in the quality control of metabolomics data. Besides the specific tools provided by the commercial software that is supplied with chromatographic MS systems (e.g., Bruker MetaboScape), other strategies and procedures are reported in the scientific literature [74].

Through GC-MS analysis using, for instance, WILEY 09 and NIST mass spectral databases, it is possible to detect hundreds of analytes, identifying them according to their retention time, molecular weight, and molecular formula. LC-MS needs a more complex approach because mass spectral databases can be compared only after data treatment. For this, common databases accessed in metabolomics studies cover a set of functions that go from data preprocessing/processing to functional analysis, metabolite identification, data integration, and visualization. The Human Metabolome Database (HMDB), MetaboAnalyst, MS-DIAL 4, MetaboLight, Metabolomics Workbench, Metlin-XCMS 3METLIN, MassBank, MetaboScape, mzCloud, FiehnLib, NIST El-MS, NIST AMDIS, The Golm metabolome database (GMD), LipidSearch, Global Natural Products Social Molecular Networking (GNPS), and Metab R are among the most widely used [75]. Some of this software is free to use, such as MZmine 4 [76] and Sirius [77].

The complex matrices that are obtained from data acquisition can be elaborated by several statistical methods. Univariate and bivariate statistics can be applied when one or two variables are evaluated (t-test, fold change, Mann–Whitney, Wilcoxon test, ANOVA, regression, etc.), and thus have limited use in metabolomics. Multivariate statistics, applied when many variables are measured, are of more help, but it is more complex and sometimes require dimensional reduction methods. Multivariate statistics can be performed following the unsupervised and supervised methods: unsupervised analyses summarize variations in the data (without regard to the response) and supervised analyses assess the variables to find the combination that best explains a causal relationship. The multivariate analysis techniques most commonly used in metabolomics studies for compound characterization, pattern recognition, fingerprinting, and biochemical marker detection include principal component analysis (PCA), hierarchical cluster analysis (HCA), partial least-squares regression (PLS), and orthogonal partial least-squares (OPLS), support vector machine (SVM), and K-nearest neighbors (KMN) algorithm. A useful tool like PLS-DA should be carefully evaluated because is susceptible to over fitting by producing patterns of separation, even for data randomly drawn from the same population. Recently, machine learning methods have gained more and more importance. Also, for these methods, unsupervised and supervised approaches can be used. Unsupervised learning is performed on unlabeled data to discover patterns and insights without any explicit guidance or instruction. Conversely, supervised learning uses labeled datasets to train algorithms to predict outcomes and recognize patterns.

The software that can be used for processing and analyzing complex biological matrices are numerous (SIMCA^®^, IBM SPSS^®^ Statistics, MINITAB, etc.), and some of them are free to use. Sometimes, they are directly integrated with the software used for the data acquisition from the GC-MS or HPLC-MS analyses.

At this point of the data analysis workflow, the results are finally interpreted, and conclusive information of the research project is obtained. In this way, metabolomics outcomes range from honey traceability to novel bioactive compounds, from indicators of honey maturity to adulteration detection. For example, using a combined untargeted and targeted MS-based study supported by orthogonal partial least-squares discrimination analysis (OPLS-DA) and corresponding validation plot and S-plot, calycosin and formononetin were identified as chemical markers for *A. membranaceus* var. *mongholicus* Hsiao monofloral honey [9]. Additionally, Montoro et al. [11] applied PCA and PLS to establish unedone as the compound responsible for the bitter taste of *A. unedo* monofloral honey. The study and characterization of metabolites can help researchers gain more comprehensive information on the safety and efficacy of honey, especially for those with known specific markers.

### 2.5. In Vivo and In Vitro Metabolomic Approaches Exploring Honey Compound Bioactive Effects

A deeper knowledge of the metabolic transformation of honey compounds through in vivo and in vitro studies can clarify the metabolic pathways and pharmacokinetics of specific compounds and allow for speculation on their potential health applications.

Yin et al. [78] studied the metabolites of acacetin in rats, both in vivo (plasma, bile, urine, and feces) and in vitro (liver microsomes), utilizing UHPLC-Q-TOF-MS/MS. Acacetin is a flavone typical of acacia honey and, in the rats, it undergoes oxidation, loss of CH_2_, reduction, hydrolysis, glucuronide conjugation, sulfate conjugation, methylation, and N-acetylation. For the detected metabolites obtained in phases I and II, metabolic pathways of acacetin in vivo and in vitro were also proposed [78], showing how metabolomics approach can help to understand if new metabolites can have an impact on honey consumption. Idriss et al. [65] investigated the antiproliferative effects of raw and powdered manuka honey on different cancer cell lines (human and murine) and indicated the inhibition of tumor cell growth by both types of samples tested.

Metabolomics can elucidate toxic compounds in honey. For instance, 1,2-unsaturated pyrrolizidine alkaloids (PAs) are plant-derived metabolites that can be found in honey from different *Senecio*, *Eupatorium*, *Tussilago Echium*, *Heliotropium*, *Symphytum*, and *Trichodesma* species. The double bond between C_1_ and C_2_ makes these compounds toxic because they exhibit strong hepatotoxic, genotoxic, cytotoxic, neurotoxic, and tumorigenic potentials [79]. This is due to the metabolic cleavage of the double bonds that generates radicals that can cause severe liver damage through a process involving microsomal P450 (CYP). This occurs because the catalytic cytochrome P450 cycle involved in the toxification of 1,2-unsaturated PAs produces metabolites that form adducts with proteins and DNA. Treatment with senecionine, a PA able to induce an acute toxic model on rats, demonstrated, after LC-QTOF MS and OPLS-DA S-plot, that the bile acid metabolism pathway is strictly associated with senecionine-induced hepatotoxicity [80].

## 3. Conclusions and Future Perspectives

The application of metabolomics to honey samples can be an efficient strategy to ensure the traceability and authenticity of honey, identify its active metabolites, and discover potential chemical biomarkers and their formation and accumulation, which is particularly useful for bioorganic research. Untargeted (i.e., analysis of all detectable metabolites), suspect, and targeted (i.e., analysis of selected subsets of metabolites) metabolomics studies can be performed. Metabolites of interest such as maturity biomarkers [4], some rare compounds with significant pharmacological potential (e.g., 1-fluoro-4-methylbenzene) [7], some metabolites that have been reported as honey constituents for the first time (e.g., dehypoxanthine futalosine), or some metabolites or metabolite classes that are not yet sufficiently investigated, e.g., cyclitols [29], may be traced, suspected/verified, or targeted by metabolomics approaches over diverse honey varieties. Investigating the chemical diversity of honey, identifying key metabolites, and tracing their sources may serve as a useful basis for future metabolomics research in bioorganic studies, especially for understudied types of honey, and open up new sources of key active metabolites. Some rare types of honey, such as honey from *Schefflera octophylla* (Lour.) Harms and other as-yet unstudied honeys included in this review pave the way for future research to uncover the key metabolites and the complex processes of their formation. Melissopalynological analysis, when incorporated into metabolomic studies, appears in a new light as a valuable tool that helps in tracing plant sources that contribute to the metabolic profile of honey, especially those with biologically active metabolites of potential medicinal importance. In addition, other honey-associated sources (e.g., microorganisms) of bioactive metabolites discovered by metabolomics may be of interest for the biotechnology of specific key metabolites. Honey presents a challenge for research due to its variability and complexity, which necessitates the development of new appropriate, simple, and environmentally friendly sample preparation methods. Some other methods that are already applied to honey and could be promising should be included and compared with the methods commonly used in metabolomics research. The importance of metabolomics research for potential innovations in honey characterization and unlocking the full bioorganic potential of honey is exceptional. Future directions in the field of bioorganic research of honey can be summarized and outlined, including (i) integrating more advanced data processing tools and conducting large-scale biomarker validation studies, (ii) addressing the mechanism of biomarker formation, (iii) conducting metabolomics research on understudied honey types and the application/development of green methods for honey sample preparation, and (iv) investigating the influence of different honey varieties as a food source, especially those with specific enhanced biomarkers, on metabolic pathways and health.

## Figures and Tables

**Table 1:** Examples of key metabolites identified by GC-based metabolomics in different honey types.

Key Metabolite	Chemical Structure	Honey Sample Description	Significance of Metabolite	Metabolomic Strategy	Reference
3,4,6,6-tetramethylbicyclo [3.2.1]oct-3-ene-2,8-dione	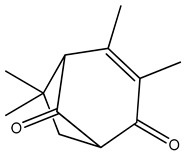	*Arbutus* (*Arbutus unedo* L., *Ericaceae*) honey (strawberry tree honey) from Greece	aroma compound pharmaceutical honey volatile metabolite (PHVM)	non-targeted metabolomic methodology HS-SPME-GC-MS + implementation of the honey code + analysis of variance (ANOVA)	[43]
*trans*-beta-ionone-5,6-epoxide	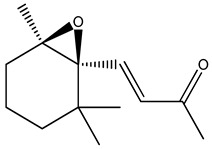	*Citrus* sp (*Murcott tangerine* L. and *Jaffa orange* L.) honey from Egypt	present in citrus worker honey bee and its honey	detailed metabolomic analysis HS-SPME-GC-MS + multivariate analysis (MVA)	[5]
sulfonylbis-metahane	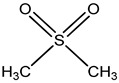	polyfloral commercial honey from Botswana, southern African region	pharmacologically active organosulfur compound	untargeted metabolomics GC–MS/SPME + AMDIS/Metab R data processing + database search + MINITAB + partial least squares (PLS) analysis	[7]
8-hydroxyoctanoic acid	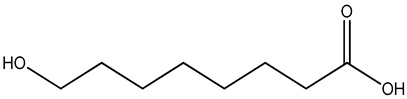	*Apis cerana cerana* (*A. cerana*) and *Apis mellifera ligustica* (*A. mellifera*) honey	markers for accurate identification of honey entomological origin	targeted quantification combined with multivariate statistical analysis	[52]
3,10-dihydroxydecanoic acid	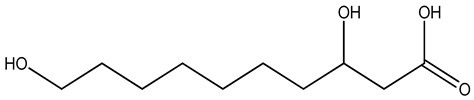				
1-nonanol	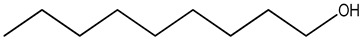	*A. cerana* honey	volatile odor species markers for *A. cerana* honey odor compounds discriminating honey from *A. cerana* and *A. mellifera*	untargeted and targeted metabolomics approaches HS-GC-IMS untargeted analysis + principal component analysis + orthogonal partial least-squares discrimination analysis GC-MS identification and confirmation of markers targeted quantitative analysis of markers by HS-SPME-GC−MS untargeted and targeted metabolomics approaches HS-GC-IMS untargeted analysis + principal component analysis + orthogonal partial least-squares discrimination analysis GC-MS identification and confirmation of markers targeted quantitative analysis of markers by HS-SPME-GC−MS	[10]
1-heptanol	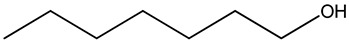				
phenethyl acetate	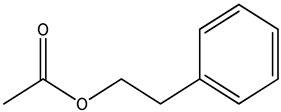				
benzaldehyde	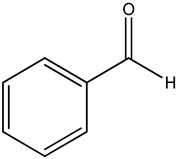	*A. mellifera* honey	volatile species markers for *A. mellifera* honey odor compounds discriminating honey from *A. cerana* and *A. mellifera*		
heptanal	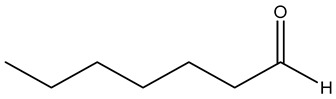				
phenylacetaldehyde	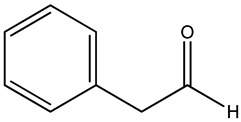				
gluconic acid	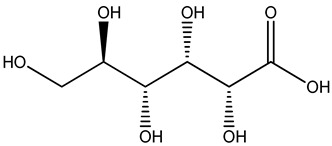	stingless bee honey	metabolites related to antioxidant activity	GC-MS analysis + data (pre)processing + orthogonal partial least square (OPLS) model	[30]
pinitol	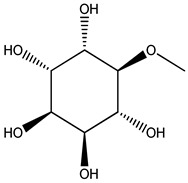				
mannitol	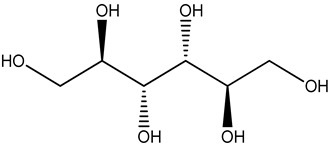				
myo-inositol	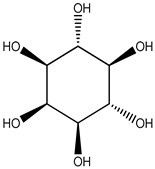				
nicotinealdehyde	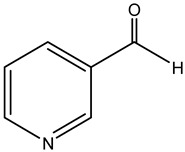	red raspberry (*Rubus idaeus* L.)	compound correlated with the pollen count of *Rubus idaeus* L.	melissopalynological analyses concurrently performed with untargeted SPME-GC–MS and HPLC-Orbitrap metabolomic + hierarchical cluster analysis (HCA) and multidimensional scaling (MDS) multivariate analysis	[18]

**Table 2:** Examples of key metabolites identified by HPLC-based metabolomics in different honey types.

Key Metabolite	Chemical Structure	Honey Sample Description	Significance of Metabolite	Metabolomic Strategy	Reference
calycosin	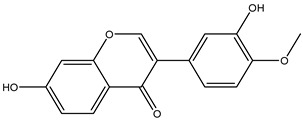	traditional Chinese herbal medicine *Astragalus membranaceus* var. *mongholicus* Hsiao	markers for monofloral honey from *Astragalus membranaceus* var. *mongholicus* Hsiao	combined untargeted and targeted UPLC-Q-TOF-MS/MS + correlated analysis by orthogonal partial least-squares discrimination analysis (OPLS-DA) model	[9]
formononetin	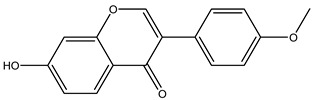				
N_1_, N_5_, N_10_-(*E*)-tricoumaryl spermidine	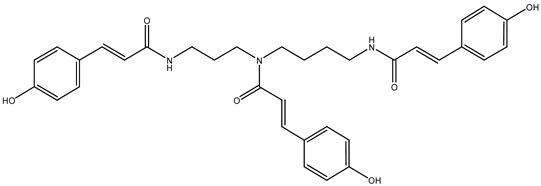	*Triadica cochinchinensis* honey (TCH)	plant-derived characteristic compound in TCH	UPLC-QTOF-MS targeted and untargeted metabolomics analyses	[58]
safflomin A	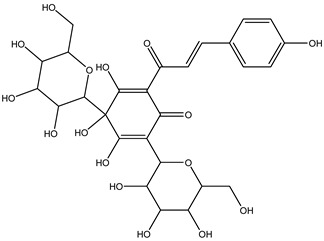	medicinal plant *Carthamus tinctorius* L.	a novel chemical marker for *Carthamus tinctorius* L. (safflower) monofloral honey	ultrahigh-performance liquid chromatography/quadrupole time-of-flight mass spectrometry (UHPLC/Q-TOF-MS) + partial least-squares discriminant analysis (PLS-DA)	[59]
3α-Hydroxylup-20(29)-ene-23,28-dioic acid (HLEDA)	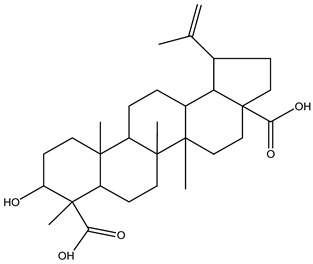	*Schefflera octophylla* (Lour.) Harms	phytogenic chemical marker for monofloral honey from *Schefflera octophylla* (Lour.) Harms authentication	ultrahigh-performance liquid chromatography—quadrupole time-of-flight tandem mass spectrometry (UHPLC-Q-TOF-MS/MS)-based untargeted metabolomics + targeted ultrahigh-performance liquid chromatography—triple quadrupole mass spectrometry (UHPLC-QqQ-MS/MS)-based method	[61]
methyl 3- aminobenzoate (M3AB)	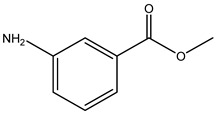	“Anama” honey: monofloral honey produced from the nectar of the *Erica manipuliflora* plant, a heather bush of the Greek island of Ikaria	potential chemical marker of Anama honey	untargeted ultrahigh-performance liquid chromatography–hybrid quadrupole orbitrap high-resolution mass spectrometry (UHPLC-HRMS) profiling + principal component analysis (PCA), orthogonal projections to latent structures discriminant analysis (OPLS-DA), and differential analysis	[16]
4- ethylbenzaldehyde	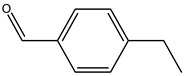		discriminating chemical compound of Anama honey detected for the first time in Anama heather honey		
dehypoxanthine futalosine	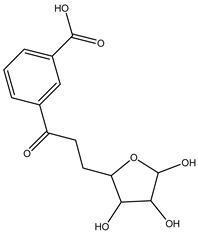		first reported as a potential honey constituent		
ganolucidic acid B	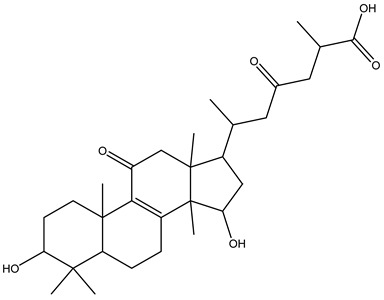		first report in honey		
domesticoside	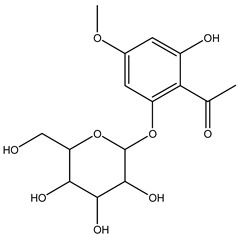		rarely reported compound putatively annotated for the first time in Anama honey		

## Data Availability

No new data were created or analyzed in this study. Data sharing is not applicable to this article.
